# Enhancement of Cellular Adhesion and Proliferation in Human Mesenchymal Stromal Cells by the Direct Addition of Recombinant Collagen I Peptide to the Culture Medium

**DOI:** 10.1089/biores.2019.0012

**Published:** 2019-11-22

**Authors:** Koji Muraya, Tomoyuki Kawasaki, Takeshi Yamamoto, Hidenori Akutsu

**Affiliations:** ^1^ Bioscience and Technology Development Center, FUJIFILM Corporation, Kanagawa, Japan.; ^2^ Department of Reproductive Medicine, National Center for Child Health and Development, Tokyo, Japan.

**Keywords:** ECM, MSC, recombinant peptide

## Abstract

Mesenchymal stromal cells (MSCs) have considerable potential for a wide range of clinical applications and regenerative medicine and cell therapy. As a consequence, there is considerable interest in developing robust culture methods for producing large number of MSCs for use in repair of injured tissues or treatment of diseases. In general, tissue culture plates or flasks that have been precoated with substrates derived from animal tissues are used in the production of MSCs. However, these substrates can potentially cause serious problems due to contamination of the MSCs with animal-derived components. In this study, we evaluated the use of a type I collagen-based recombinant peptide (RCP) for MSC culture in an attempt to avoid the problems associated with animal cell-derived substances. This RCP is xeno free, has an increased RGD (Arg–Gly–Asp) sequence, and has high molecular weight uniformity. The effect of RCP on promotion of cellular adhesion and proliferation of MSCs was investigated in cultures in which RCP was included in the culture medium. The effects of RCP on promotion of cellular adhesion and proliferation of MSCs were investigated by comparing cultures in which the additive was present in the culture medium and those where the culture plates were coated with RCP. In addition, changes in gene expression profiles during cell culture were monitored by real time-polymerase chain reaction. Our analyses showed that RCP enhanced cellular adhesion and proliferation in cultures in which the additive was included in the culture medium. Our findings indicate that adding RCP to the culture medium could save time and cost in MSC culture. Our gene expression analysis indicated that RCP enhanced expression of genes encoding proteins associated with the extracellular matrix and cell adhesion.

## Introduction

Mesenchymal stromal cells (MSCs)^1^ are able to self-renew and differentiate into multilineage cells such as osteocytes, adipocytes, and chondrocytes^2^; as a consequence, MSCs have considerable potential for use in a wide range of clinical applications and are promising materials for regenerative medicine and cell therapy.^3,4^ In contrast to other stem cells, such as embryonic stem cells or induced pluripotent stem cells, MSCs offer a stable, safe, and highly accessible stem cell source. Currently, there is considerable interest in developing culture systems for the scalable expansion of MSCs to provide large number of MSCs necessary for use in the repair of injured tissues and for treatment of diseases; ∼10^8^ MSCs are needed for use on each occasion.^5^ Tissue culture plates or flasks precoated with substrates such as fibronectin (FN) or laminin (LAM) are generally used to generate sufficient MSCs; however, these coating substrates are derived from animal tissues and so raise the problems of contamination of the obtained MSCs and of differences among lots.

In a previous study, we developed a type I collagen (COL)-based recombinant peptide (RCP) for use in cell culture.^6^ This peptide has an increased RGD (Arg–Gly–Asp) sequence, is xenogeneic-free, and has a highly uniform molecular weight. The peptide is biodegradable and biocompatible. In this study, we describe a novel culture system for MSCs that employs RCP using conventional culture conditions. Our analyses show that inclusion of RCP in the culture medium enhances cellular adhesion and proliferation. In addition, we show that expression of genes encoding proteins associated with the extracellular matrix (ECM) and cell adhesion is elevated in cultures including RCP. The inclusion of RCP in the culture medium obviates the need to coat culture vessel; thus, our new culture approach reduces processing time and leads to a reduction in time and cost of MSC culture. The xeno-free substrate RCP can support more efficient and safer scalable culture systems for production of MSCs.

## Materials and Methods

### Cell culture

Three MSC lines were used in this study: human bone marrow stem cells (HMSCs) from Lonza (PT-2501); human epiphyseal chondrocyte (HEC)^7^ cell lines established from cartilage of hyperdactylia at the National Center for Child Health and Development (NCCHD); and an upper limb bone marrow cell (UBMC) line established at NCCHD. For the latter two cell types, parental written informed consent was obtained and their use in this study was approved by the Institutional Review Board of the NCCHD.

HMSCs, HECs, and UBMCs were cultured in low-serum concentration (2%) medium (Mesen PRO RS^®^; Thermo Fischer Scientific, MA) and in serum-free medium (PRIME-XV^®^ MSC Expansion XSFM; FUJIFILM Irvine Scientific, CA) at 37°C in air. The cells were cultured on Cellnest^TM^ (FUJIFILM Wako Pure Chemical Corporation, Tokyo, Japan); Cellnest is a solution containing 0.1% type I COL-based RCP. RCP has a repetitive RGD sequence designed to enhance cellular adhesion.^8^

As a control, RCP-coated culture plates were prepared: 0.1% RCP solution (2 mL; Cellnest) was dispensed onto the culture dishes at 37°C. After 2 h, the RCP solution was removed from each well and Dulbecco's phosphate-buffered saline (DPBS) was added to each well. The plates were kept at room temperature for 30 min, and the DPBS was then removed.

### Cell adhesion assay

Various methods are available to evaluate cell adhesion.^9^ In this study, we evaluated the effect of RCP on cell adhesion under different culture conditions, with RCP and without RCP in the medium using time lapse images recorded in a BioStation CT system (Nikon, Tokyo, Japan). The medium (1 mL) was dispensed into the culture plate (12-well; Falcon TC plate, Corning, NY) and RCP solution (0.1%) was added directly into the medium so that the RCP concentration was in the range 0.028–28 μg/mL. The rate of cell adhesion was calculated from number of cells in every 3 min by counting the bright spot of each image.

### Cellular growth

HMSCs (0.3 × 10^6^ cells/well) were seeded into a six-well Falcon plate in 2 mL Mesen PRO medium. Three RCP concentrations (0.028, 2.8, and 28 μg/mL) were prepared and added to the culture medium; these concentrations were based on the amount of RCP (2.8 μg/mL) used to coat wells. Control cultures received the same volume of DPBS in place of the RCP solution. Cells were cultured and passaged every 7 days. At the end of the culture period, cells were collected using trypsin-ethylenediaminetetraaceticacid (Lonza, Basel, Switzerland) and were counted using a Vi-Cell XR cell counter (Beckman Coulter, Inc., CA). Three repeat cultures were assessed for each treatment condition, and cell proliferation curves were obtained. Population doubling (PD) at the *n*th passage (*P*^n^) can be described as follows:

PD at *P*^n^ = PD at *P^n^* ^− 1^ + log_2_ (counted cells/seeded cells).

### PCR analysis

After fluorescence-activated cell sorting, RNA was isolated from freshly isolated c-kit^+^ cells and from control cardiomyocytes using an optimized version of the TRIzol (Invitrogen, CA) protocol to increase precipitation time in isopropanol. RNAs were pooled from two independent cell sorts to accumulate 1000 ng of total RNA and were assigned RNA Integrity Number values using an Agilent 2100 bioanalyzer. cDNAs were synthesized using the RT2 First Strand Kit and the RT2qPCR Master Mix according to the manufacturer's instructions (Qiagen-SABiosciences, Hilden, Germany). The cDNAs were placed into PCR array plates and amplified as described hereunder (“Real-time quantitative PCR”). Data were analyzed using the RT^2^Profiler^TM^ PCR Array Data Analysis Template available on the manufacturer's website.

### Extraction of mRNA

At the end of the culture period, the cells were collected and counted. The cells were then stored until required in a freezer at −80°C. mRNA was extracted from the cells using an RNeasy Plus MiniKit and QIA Shredder (both from Qiagen). After extraction, the mRNA was stored in a freezer at −80°C. The quantity and purity of the extracted mRNA were determined by measuring absorbance at 260 and 280 nm using a NanoDrop ND-1000 spectrophotometer (Thermo Fischer Scientific, Inc.). The stored mRNA was used to prepare cDNA as soon as feasible after collection.

### Preparation of cDNA

Extracted mRNA (10 μL), primer (1 μL), and dNTPs (1 μL; Thermo Fischer Scientific, Inc.) were mixed in a reaction tube and heated at 65°C for 5 min using the thermal cycler ProFlex^TM^ PCR System (Life Technologies) to anneal the primer to the template RNA. Then, 5 × FS buffer (4 μL), 0.1 M dithiothreitol (2 μL), RNase OUT^TM^ (1 μL, RNase Inhibitor; Invitrogen), and SuperScript IV Reverse Transcriptase (1 μL; Invitrogen) were added to the reaction tube and incubated first at 25°C for 10 min, at 50°C for 50 min, and then at 85°C for 5 min; finally, the reaction temperature was lowered to 4°C. Residual RNA was removed by adding RNaseH (1 μL, *Escherichia coli* Ribonuclease H; Invitrogen) and incubating at 37°C for 20 min. The cDNA was assessed by measuring absorbance at 260 and 280 nm using a NanoDrop ND-1000 spectrophotometer and preserved at −80°C in a freezer.

### Gene expression profile analysis

Gene expression profiles in the cell cultures were analyzed by real time-polymerase chain reaction (RT-PCR) using RT^2^ Profiler^TM^ PCR Arrays (Human Extracellular Matrix & Adhesion Molecules; Qiagen) and RT^2^ SYBR^®^ Green qPCR Mastermix (Qiagen). The RT-PCR analysis was performed using a Quant Studio^TM^ 7 Flex RealTime PCR System (Expression Suite Software 1.0.4; Life Technologies). A reaction mixture containing cDNA (102 μL), RT^2^ SYBR Green qPCR Mastermix (1350 μL), and Ultra Pure Water (1248 μL; Invitrogen) was prepared and 25 μL was dispensed into each well of the RT-PCR array. Then, RT-PCR array was set to Quant Studio^TM^ 7 Flex RealTime PCR System and started to analyze. PCR amplification was performed using the manufacturer's protocol for the Quant Studio 7 Flex RealTime PCR System. Semiquantitative analysis was performed to compare relative levels of expression using *ACTB*, *B2M*, *GAPDH*, *HPRT1*, and *RPLP0* as the housekeeping genes.

### Statistical analyses

Three repeat cultures were assessed for gene expression analysis, and three RCP concentrations (0.028, 2.8, and 28 μg/mL) were prepared and added to the culture medium; control cultures received the same volume of DPBS in place of the RCP solution. A *p*-value <0.05 was considered statistically significant.

## Results

### RCP facilitates cell adhesion

**FIG. 1.**
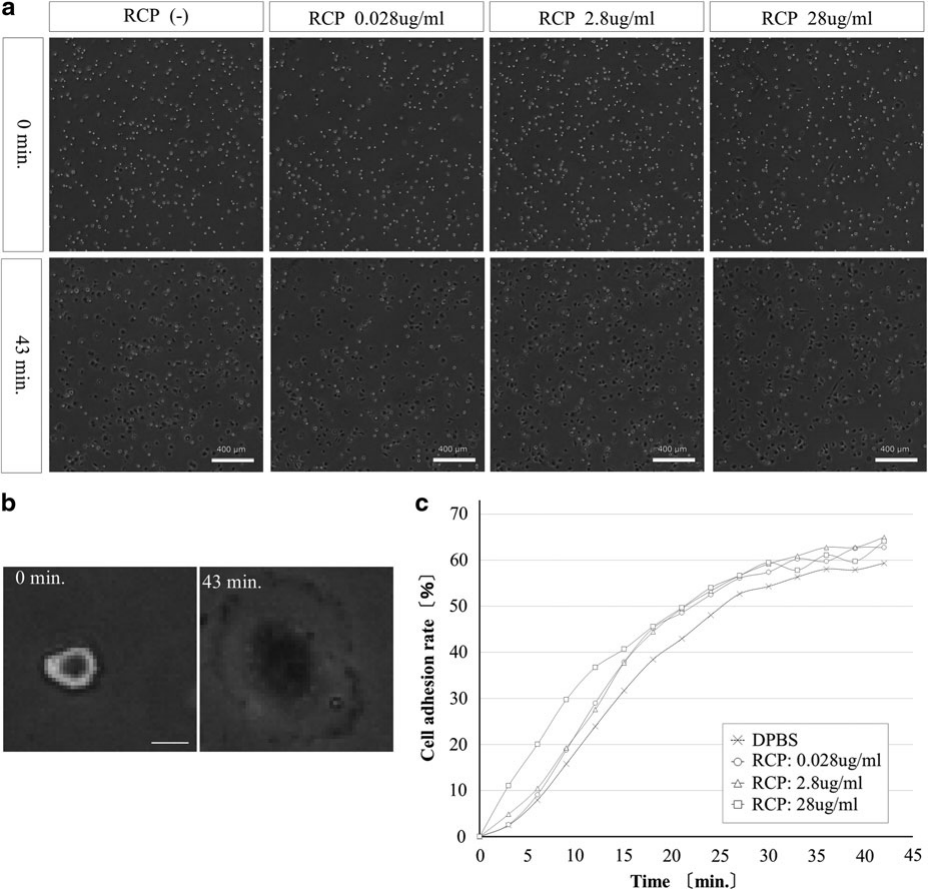
Effect of RCP to cell adhesion. **(a)** The cell adhesion images to the surface of cell culture plate. Before adhesion, cells have a bright appearance, after adhesion, they appear darker. Bar = 400 μm. **(b)** Adhering cell is darker image. Bar = 20 μm. **(c)** Relationship between the cell adhesion rate and the concentration of RCP in low serum medium. RCP, recombinant peptide.

As already described, we analyzed cell adhesion to the surface of the wells using a BioStation CT system (Nikon). Before adhesion, cells have a bright appearance; after adhesion, they appear darker (Fig. 1a, b and Supplementary Video S1). The rate of attachment of the cells can be estimated using the following formula:

Cell adhesion rate (%) = 1 − (number of bright spots at each interval/number of bright spots at 0 min) × 100.

**FIG. 2.**
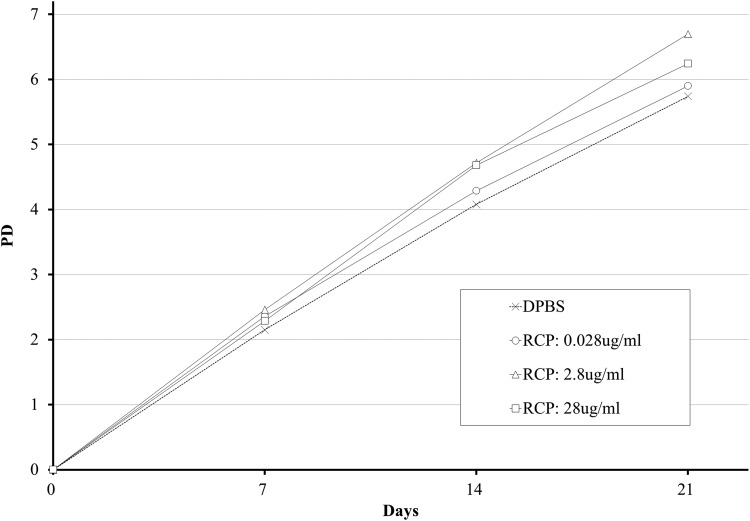
Cellular growth by direct addition of RCP. Growth curve of HMSC (Lonza) (medium: Mesen PRO). As a control, DPBS is added instead of RCP. Dose dependence of RCP is examined in the range from 0.028 to 28 μg/mL. DPBS, Dulbecco's phosphate-buffered saline; HMSC, human bone marrow stem cell.

Three biological replicates were performed for the cell adhesion assays. Various concentrations of the RCP addition in the medium could support initial attachment of the MSCs, there are no significant differences though. By comparison with cultures in which the RCP was not included in the medium, the rate of cell adhesion increased in cultures using medium with added RCP; however, the increases were not significant at any of the tested concentrations of RCP (Fig. 1c). Interestingly the effect of the RCP was relatively larger in serum-free medium (Supplementary Fig. S1). To compare differences in cellular proliferation in cultures using RCP-coated plates and those using RCP in the culture medium, we examined the cell proliferation curves from both conditions. It is likely that the simple addition of RCP to the medium supported proliferation of MSCs (Fig. 2 and Supplementary Fig. S2).

### Gene expression profiles

The effects of RCP in the culture medium on gene expression were analyzed using an RT-PCR array.

HMSCs (0.4 × 10^6^ cells/dish) were cultured in Mesen PRO RS medium (8 mL) in 90 mm dishes (Sumitomo Bakelite Co., Ltd., Tokyo, Japan) with or without RCP (concentration range 0.028–28 μg/mL); three biological replicates were performed for each culture condition. The cells were cultured for 7 days.

**FIG. 3.**
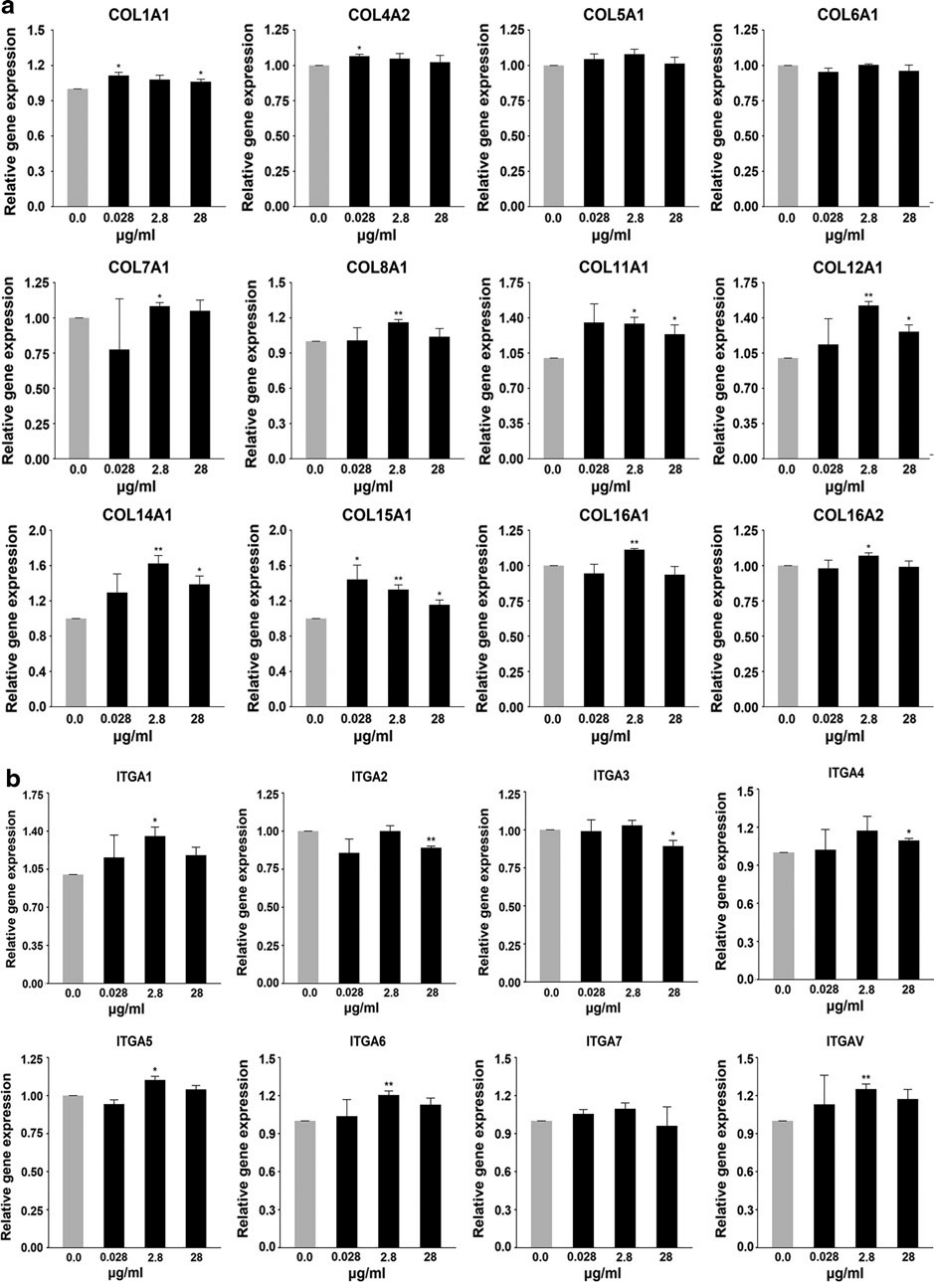

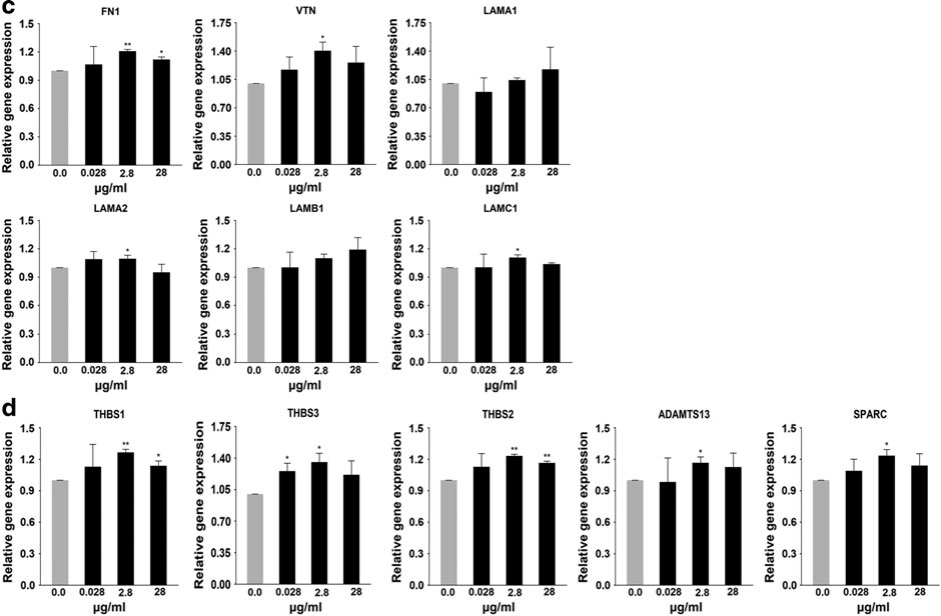
ECM and cell adhesion molecule-associated gene expression profile in HMSC culture of RCP direct addition. **(a)** Collagens. **(b)** Cell adhesion-associated molecules. **(c)** Other ECM and cell adhesion-associated molecules. **(d)** Other molecules. ECM, extracellular matrix.

In cultures using medium including RCP, some genes showed increased expression, such as the ECM genes for COLs and the cell adhesion-associated gene vitronectin (*VTN*). The levels of expression of the enhanced genes increased with RCP concentration (Fig. 3).

## Discussion

The effect of the inclusion of type I COL-based RCP on promotion of cellular adhesion and proliferation of MSCs was investigated. It is likely that the simple addition of RCP to the medium supported attachment and proliferation of MSCs; however, the relationship between cell adhesion and proliferation remains unsolved in these results. With regard to possible mechanisms by which RCP might promote cell proliferation and cell adhesion, our analysis of an RT-PCR array of ECM and cell adhesion molecules indicated that the presence of RCP in the culture medium enhanced expression of genes encoding ECM proteins, and of those associated with ECM remodeling and cell adhesion. Although the differences of those gene expression levels are rather subtle, some genes show that the level of expression of them increased with the concentration of RCP.

The ECM is a major component of the microenvironment of a cell and participates in most basic cell behaviors; ECM remodeling is an important process for cell proliferation.^10,11^ ECM binds ligands and creates concentration gradients for many growth factors and signal transduction materials, such as fibroblast growth factors, transforming growth factor (TGF)β, and Wnts. ECM degradation can also release important signaling molecules. Cell signaling through the ECM can affect cell fate decisions, cell proliferation, and survival.^12,13^

Our results showed that the expression of some COL genes was enhanced by the inclusion of RCP in the culture medium (Fig. 3a). COL is one of the structural constituents of the ECM and promotes MSC adhesion, survival, and proliferation.^14^ Cell adhesion on COL-coated plates is high and occurs within a short period of cell seeding (2 h); no increase in cell adhesion is seen at 12 h. These observations are in agreement with our data given in this study. COL also protects MSCs from oxidative and nutrient stress-induced cell death that might occur *in vivo* during ischemia.^15^ This suggests that culturing MSCs with RCP increases cell proliferation and promotes survival from stress-induced cell death due to the enhancement of *COL* gene expression. Furthermore, the enhancement of *COL* expression by RCP could lead to active cell migration and cell attachment with more cell-to-cell surface contact points.

Integrins are known mediators for cell–ECM interactions as they have receptors for the RGD motifs of LAM and FN. LAM and FN are essential for the formation of the basement membrane in collaboration with other proteins. The fate of MSCs is believed to be largely determined by biochemical and mechanical cues from the ECM that are sensed and transmitted by integrins; specific ECM constituents influence MSC proliferation and differentiation.^16^ For example, knockdown of integrin-alpha-5 *(ITGA5)*/integrin-alpha-V *(ITGAV)* results in an increase in differentiation.

MSCs interact with the surrounding microenvironment mainly through integrins. The culture of MSCs on FN or RGD peptide-coated plates promotes proliferation but not differentiation; proliferation of primary MSCs is favored by the presence of compounds containing RGD motifs, such as FN or VTN.^17^ Signals from these ECM constituents are mainly recognized by ITGA5 and ITGAV. In this study, cell proliferation might have been promoted by the increase in expression of integrin genes such as *ITGA5* or *ITGAV* (Fig. 3b).

VTN and LAM are associated with cell adhesion and with the ECM. There is evidence that VTN and COL type I can promote osteogenic differentiation in MSCs, whereas LAM stimulates the proliferation of MSCs but suppresses chondrogenesis.^18,19^ In this study, expression of both *VTN* and *LAM* genes was enhanced by the addition of RCP to the culture medium (Fig. 3c). Although we did not confirm that RCP could promote osteogenic differentiation by RCP, such an activity is feasible as *VTN* expression was enhanced because RCP is a COL type I molecule.

The thrombospondin (THBS) protein family is composed of secreted glycoproteins; THBS2 and THBS3 are components of the ECM. MSCs have a unique tropism for wounded tissue, a high differentiating capacity, the ability to induce tissue repair, and have anti-inflammatory and immunoregulatory activities. THBS1 is a major regulator of MSCs and induces MSC proliferation. This proliferation is mediated by THBS1-induced activation of endogenous TGFβ, and it cannot activate TGFβ in the absence of THBS2 activity.^20^ The ADAMTS (a disintegrin and metalloproteinase with THBS motifs) enzymes are secreted, multidomain matrix-associated zinc metalloendopeptidases that have diverse roles in tissue morphogenesis and pathophysiological remodeling in inflammation and in vascular biology.^21^ Our results show that expression of genes associated with MSC proliferation and adhesion was enhanced by addition of RCP to the culture medium; moreover, expression of genes associated with tissue repair or anti-inflammatory activities, such as *THBS* and *ADAMTS*, was also enhanced (Fig. 3d).

## Conclusion

The type I COL-based RCP would be effective at promoting cellular adhesion and proliferation of MSCs simply by direct inclusion in the culture medium. Comparison of the outcomes of culturing MSCs in RCP precoated plates with that of culture in medium containing RCP at the same concentration (2.8 μg/mL) showed that promotion of cell proliferation was similar in both. Moreover, both of these cell culture conditions would be more effective than cultures on uncoated plates or in medium lacking RCP. Regarding the direct addition of RCP in the medium, including the same amount of RCP to precoating (2.8 μg/mL) was obtained best results in cell proliferation. The promotion of cell proliferation was maximum at an RCP concentration of 2.8 μg/mL; higher concentrations of RCP did not induce a greater effect. ECM remodeling was highly activated during MSC adhesion and proliferation. Genes encoding proteins associated with cell adhesion and the ECM showed higher levels of expression as a result of ECM remodeling. Our finding that RCP promotes cellular adhesion and proliferation after inclusion in the culture medium offers a possible reduction in the cost and time for MSC culture, as it should be feasible to reduce the amount of RCP and processing time for coating wells with RCP. The coating process requires greater amounts of RCP than the simple addition of the compound to the culture medium. We also found that genes encoding proteins associated with tissue repair or anti-inflammatory activities were also enhanced by RCP. As RCP is a xeno-free compound and because there is no need to precoat wells with FN or LAM for MSC culture, the use of culture medium containing RCP would be suitable for scalable cultures of MSCs for medical applications.

## Authors Contributions

K. M. was involved in conception and design, collection and/or assembly of data, data analysis and interpretation, and article writing; T.K. contributed to collection and/or assembly of data, and data analysis and interpretation; T.Y. contributed to administrative support; H.A. was involved in conception and design, financial support, administrative support, provision of study material, data analysis and interpretation, article writing, and final approval of the article.

## Supplementary Material

Supplemental data

Supplemental data

Supplemental data

## Abbreviations Used

ACTB: actin beta
ADAMTS: a disintegrin and metalloproteinase with thrombospondin motifs
B2M: beta-2-microglobulin
COL: collagen
DPBS: Dulbecco's phosphate-buffered saline
ECM: extracellular matrix
FN: fibronectin
GAPDH: glyceraldehyde-3-phosphate dehydrogenase
HMSC: human bone marrow stem cell
HPRT1: hypoxanthine phosphoribosyltransferase 1
ITGA5: integrin-alpha-5
ITGAV: integrin-alpha-V
LAM: laminin
MSC: mesenchymal stromal cell
NCCHD: National Center for Child Health and Development
PD: population doubling
RCP: recombinant peptide
RGD: Arg–Gly–Asp
RPLP0: ribosomal protein, large, P0
TGF: transforming growth factor
THBS: thrombospondin
UBMC: upper limb bone marrow cell
VTN: vitronectin
