# Supplementary material for: Enhancement of Cellular Adhesion and Proliferation in Human Mesenchymal Stromal Cells by the Direct Addition of Recombinant Collagen I Peptide to the Culture Medium
Source: Biores Open Access. 2019 Nov 22;8(1):210–8. doi: 10.1089/biores.2019.0012 (PMC6873350; doi:10.1089/biores.2019.0012)
Supplement: Supplemental data [file Supp_Fig1.pdf]

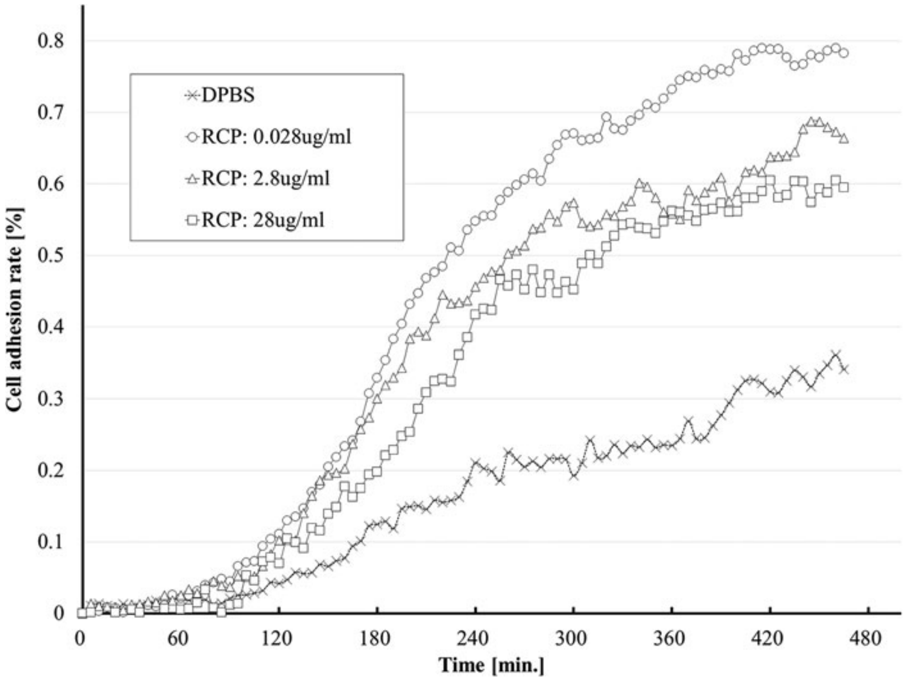

SUPPLEMENTARY FIG. S1. Effect of RCP to cell adhesion. Relationship between the cell (UBMC) adhesion rate and the concentration of RCP in serum-free medium. RCP, recombinant peptide; UBMC, upper limb bone marrow cell.
