# Supplementary material for: Enhancement of Cellular Adhesion and Proliferation in Human Mesenchymal Stromal Cells by the Direct Addition of Recombinant Collagen I Peptide to the Culture Medium
Source: Biores Open Access. 2019 Nov 22;8(1):210–8. doi: 10.1089/biores.2019.0012 (PMC6873350; doi:10.1089/biores.2019.0012)
Supplement: Supplemental data [file Supp_Fig2.pdf]

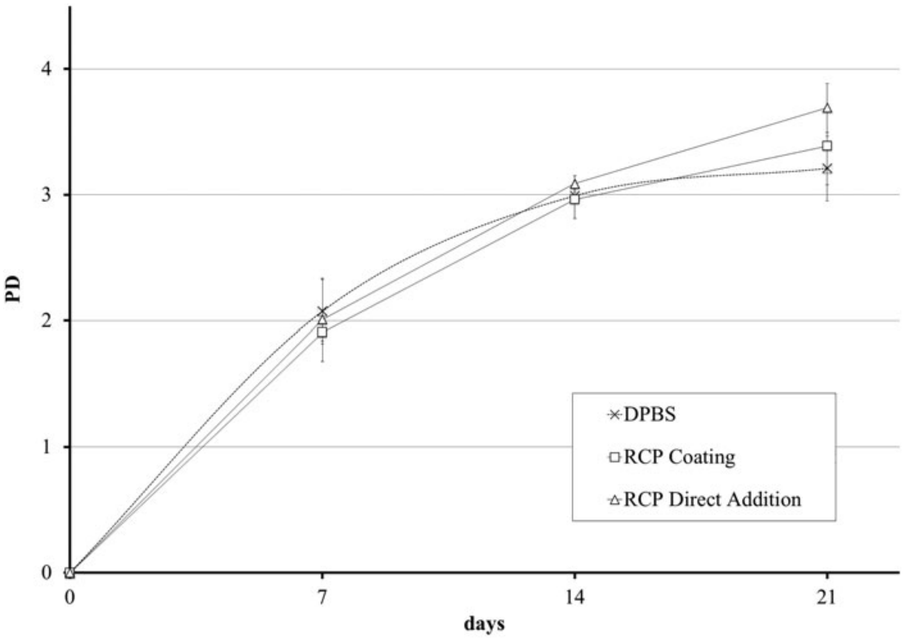

SUPPLEMENTARY FIG. S2. Cellular growth by direct addition of RCP. Growth curve of HEC (cell line Yub2540; medium: Mesen PRO). As a control, DPBS is added instead of RCP. RCP direct instillation is compared with RCP coating. Culturing MSCs in RCP precoated plates with that of culture in medium containing RCP at the same concentration (2.8 μg/mL). DPBS, Dulbecco's phosphate-buffered saline; HEC, human epiphyseal chondrocyte; MSC, mesenchymal stromal cell.
